# A new species of *Pectinaria* (Annelida, Pectinariidae), with a key to pectinariids from the South China Sea

**DOI:** 10.3897/zookeys.683.12272

**Published:** 2017-07-10

**Authors:** Jinghuai Zhang, Jian-Wen Qiu

**Affiliations:** 1 South China Sea Environmental Monitoring Center, State Oceanic Administration, Guangzhou, China; 2 Department of Biology, Hong Kong Baptist University, Kowloon, Hong Kong

**Keywords:** invertebrate, *Pectinaria*, polychaete, South China Sea, taxonomy

## Abstract

Pectinariidae is a family of polychaetes building unique ice-cream cone shaped sandy tubes. *Pectinaria torquata* **sp. n.** (Pectinariidae) is described from the coastal waters of the northern South China Sea. This new species can be distinguished from all other 25 recognized species in the genus by a combination of characters: 16 chaetigers; 26–32 cirri in the cephalic veil; 11–12 pairs of cephalic spines; uncini with major teeth arranged in two rows, each with 7–8 major teeth; presence of a dorsal posterior lobe on segments 2 and 20; 4–5 pairs of curved scaphal hooks; and an anal flap with a crenulated margin. A key to all recognized pectinariids in the South China Sea is provided.

## Introduction

Pectinariidae is a family of polychaetes commonly found in the soft bottom of coastal shallow waters. They are easily recognized by their unique ice-cream cone shaped sandy tube, and two bundles of golden thick chaetae called paleae on the first segment which they use for sediment digging (Fauchald 1977, Wolf 1984). There are 50 recognized species of Pectinariidae in five genera: 15 species of *Amphictene* Savigny, 1818; six species of *Cistenides* Malmgren, 1866; ten species of *Lagis* Malmgren, 1866; 25 species of *Pectinaria* Savigny, 1818; and four species of *Petta* Malmgren, 1866 (Hartman 1941, Hutchings and Peart 2002, Sun and Qiu 2012, García-Garza and de León-González 2014, Nishi et al. 2014, Wong and Hutchings 2015, Zhang et al. 2015). Ten species of Pectinariidae have been recorded from the South China Sea, including four species of *Amphictene*, three species of *Lagis*, and three species of *Pectinaria* (Wu and Chen 1985, Yang and Sun 1988, Paxton and Chou 2000, Sun and Qiu 2012, Salazar-Vallejo et al. 2014, Zhang et al. 2015, Glasby et al. 2016).

*Pectinaria* can be distinguished from other genera of Pectinariidae by a combination of characters: smooth opercular rim; cephalic veil free with numerous cirri; and neurochaetal uncini having major teeth arranged in two or more rows (Wong and Hutchings 2015). Here a new species of *Pectinaria* is described, based on three specimens collected from the coastal waters of the northern South China Sea.

## Materials and methods

Specimens were collected while undertaking a benthic ecology monitoring program of the South China Sea Environmental Monitoring Center (SCSEMC), State Oceanic Administration. Benthic samples were collected from the northern South China Sea using a 0.05 m^2^ van Veen grab, and rinsed through a sieve with 0.5 mm mesh size. Samples retained on the sieve were collected, fixed in 5% formalin, and later transferred to 70% ethanol. Type specimens are deposited at the Institute of Oceanology, Chinese Academy of Science (IOCAS), Qingdao. Specimens were examined under a Carl Zeiss Stemi 2000-C dissecting microscope. Morphological features were recorded using a Carl Zeiss AxioCam ICc 1 digital camera attached to the microscopes. A paratype was freeze-dried using a Xiangyi CFD-10D, gold coated using an EDT SC-150, and examined under a TESCAN CEGA 3 scanning electron microscope (SEM). Line drawings were made using a Wacom Intuos Pro Pen and Touch Large Tablet.

The taxonomic terms defined by Hutchings and Peart (2002) were used in the species description.

## Results

### Pectinariidae

Taxon classificationAnimaliaAnnelidaPectinariidae

de Quatrefages, 1866

Pectinaria Savigny in Lamarck 1818: 348

#### Type species.

*Nereis cylindraria belgica* Pallas, 1766, designated by Hartman (1959)

### Pectinaria torquata sp. n.

Taxon classificationAnimaliaAnnelidaPectinariidae

http://zoobank.org/DAFE1881-63F0-454B-922B-8E32B878B7CB

Figs 1 , 2 , 3

#### Material examined.

All type specimens are deposited in the Marine Biological Museum (MBM), Institute of Oceanology, Chinese Academy of Sciences, Qingdao

#### Holotype.

MBM240082: complete specimen, 22°45.17'N, 114°42.98'E (Daya Bay, Guangdong Province), 9.0 m water depth, muddy sand, August 2015.

#### Paratypes.

MBM240083: complete specimen, 22°35.50'N,114°33.22'E (Daya Bay, Guangdong Province), 11.0 m water depth, muddy sand, June 2015. MBM240084: incomplete specimen with 17 anterior segments, 21°39.42'N, 108°34.46'E (Beibu Gulf, Guangxi Province), 9.7 m water depth, muddy sand, August 2015.

#### Etymology.

The specific epithet *torquata* is a Latin adjective for collar, which refers to the elevated collar-like dorsal posterior lobe on segment 2, a distinctive feature for this species.

#### Diagnosis.

Opercular margin smooth. Cephalic veil free from operculum, with 26–38 cirri along the rim. Segments 2 and 20 with a dorsal posterior lobe respectively. Body with 16 chaetigers. Neurochaetal uncini with major teeth arranged in two rows. Scaphe formed by fusion of five posterior segments. Four or five pairs of scaphal hooks.

#### Description of holotype.

**Figure 1.**
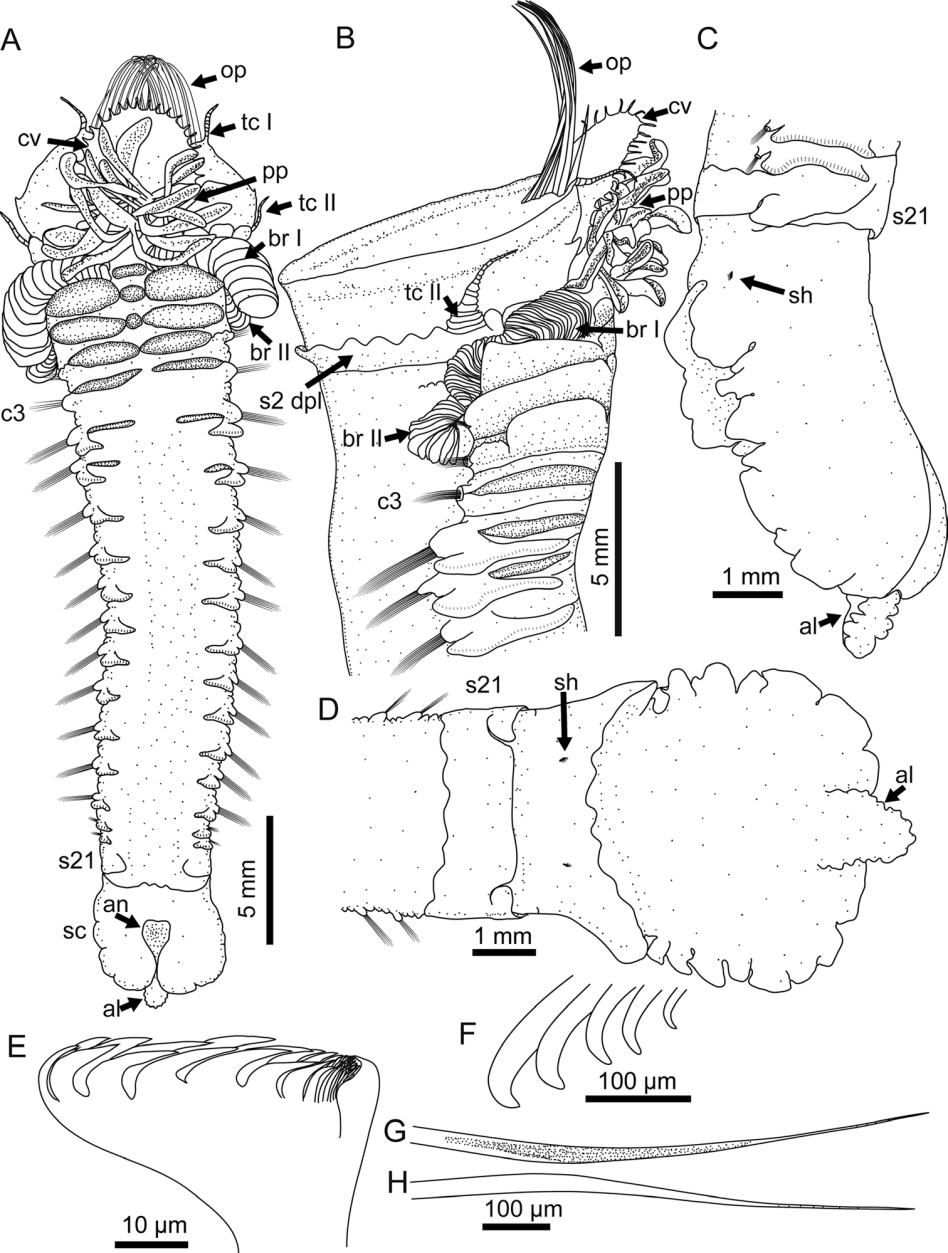
*Pectinaria torquata* sp. n. Holotype MBM240082. **A** ventral view of the whole worm **B** lateral view of anterior body **C** lateral view of the posterior including the scaphe **D** dorsal view of the posterior including the scaphe **E** an uncinus **F** scaphal hooks **G** anterior view of a notochaeta **H** posterior view of a notochaeta. Abbreviations: al, anal lobe; an, anus; br, branchia; c3 chaetiger 3; (segment 7) c16, chaetiger 16 (segment 20); cv cephalic veil; op opercular palea; pp peristomial palp; s2 dpl, dorsal posterior lobe on segment 2; s21 segment 21 sc, scaphe; sh, scaphal hooks; tc, tentacular cirrus.

Preserved specimen pale cream in color. Body stout with cephalic region enlarged (Figs 1A, 3A–B). Body length 38.5 mm including scaphe, width 9.0 mm at cephalic region. Tube straight, conical, composed of cemented sand grains and shell fragments (Fig. 3C).

**Figure 2.**
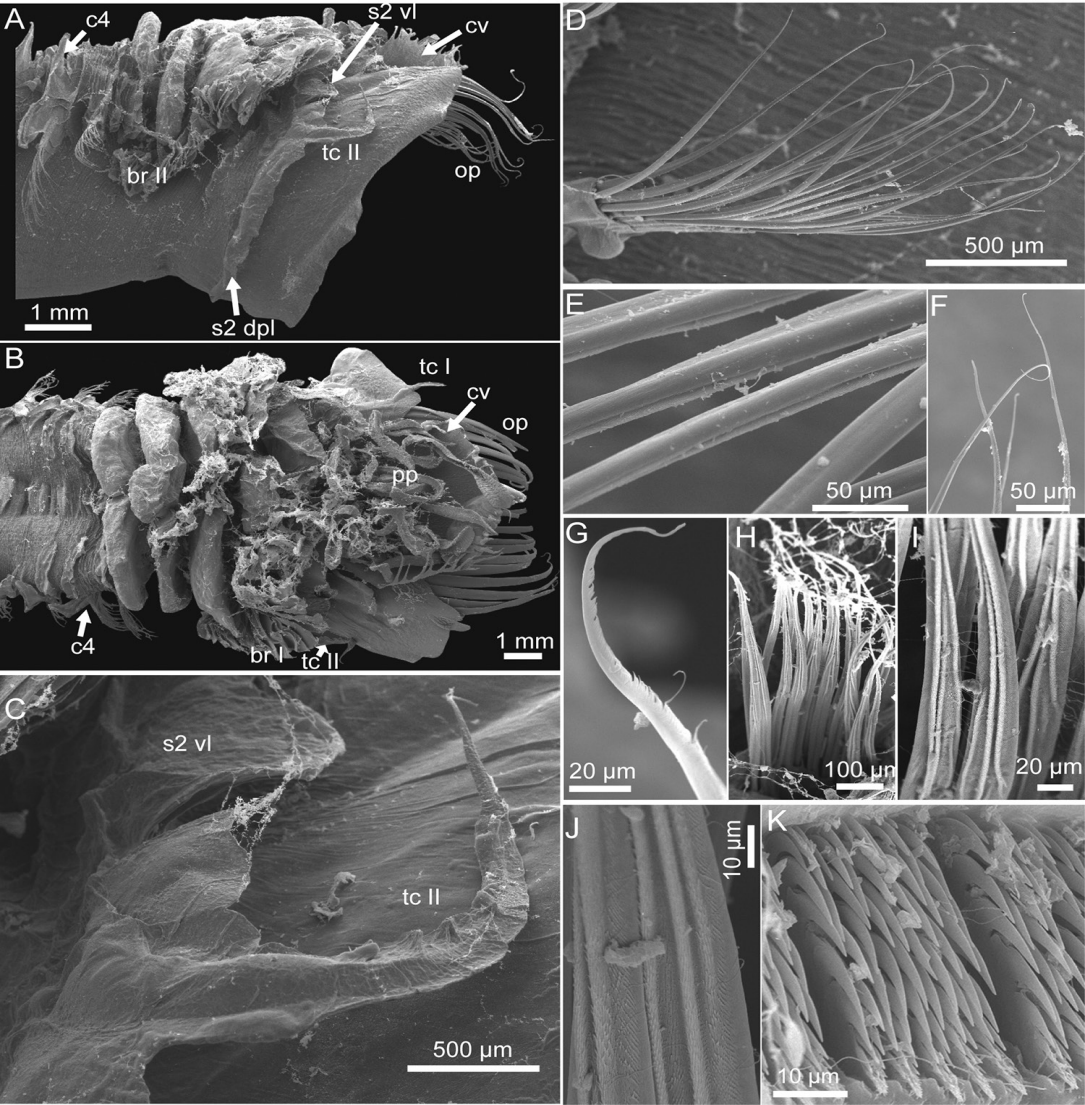
*Pectinaria torquata* sp. n. paratype MBM240084. **A** lateral view of anterior body **B** ventral view of anterior body **C** tentacular cirri II **D–F** close-up of notochaetae from chaetiger **3 G**, a notochaeta from chaetiger 5 **H–J** close-up of notochaeta from chaetiger 12 **K** uncini from chaetiger 12 Abbreviations: br, branchia; c4, chaetiger 4 (segment 8); cv, cephalic veil; op, opercular palea; pp, peristomial palp; s2 dpl, dorsal posterior lobe on segment 2; s2 vl, ventral lobe on segment 2; tc, tentacular cirrus.

**Figure 3.**
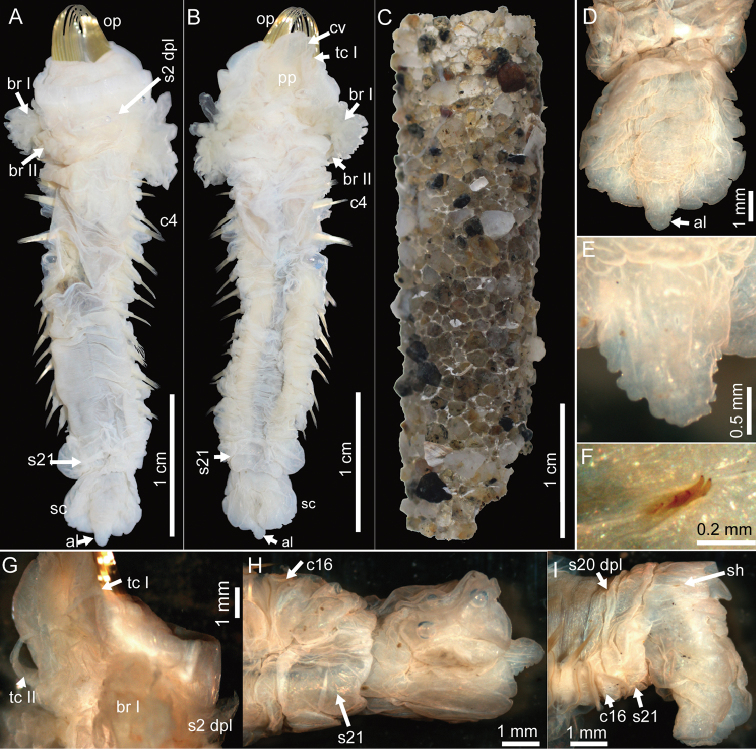
*Pectinaria torquata* sp. n., holotype MBM240082. **A** dorsal view of whole specimen **B** ventral view of whole specimen **C** tube **D** dorsal view of posterior end **E** anal flap **F** scaphal hooks **G** lateral view of anterior end **H** ventral view of posterior end. Paratype MBM240083 **I** lateral view of posterior end. Abbreviations: al, anal lobe; br, branchia; c4, chaetiger 4 (segment 8); c16, chaetiger 16 (segment 20); cv, cephalic veil; op, opercular palea; pp, peristomial palp; s2 dpl, dorsal posterior lobe on segment 2; s20 dpl, dorsal posterior lobe on segment 20; s21, segment 21; sc, scaphe; sh, scaphal hooks; tc, tentacular cirrus.

*Opercular margin* raised with smooth edge (Figs 1A–B, 2A, 3A–B). Cephalic veil free from operculum on dorsal side, with 26 cirri distributed along the rim (Figs 1A–B, 2A–B). Operculum with two bundles of paleae, each bundle with 12 stout, flattened, golden bristles curved dorsally and tapering to pointed tip (Figs 1A–B, 2A–B, 3A–B).

*Segment* 1 with pair of tentacular cirri arising from antero–ventral edge near outer most paleae. Segment 2 with pair of tentacular cirri arising from both sides; tentacular cirri connected by flattened ridge running across venter (Figs 1B, 2A–C), and dorsal posterior lobe running across both sides and dorsum (Figs 1B, 2A, 3A, 3G). Segments 3 with pair of comb-like lateral branchiae, and a median ventral lobe (Figs 1A–2, 2B, 3B). Segments 4 with pair of comb-like lateral branchiae which are smaller than the branchiae on segment 3, a small medial ventral lobe and two large ventral lateral lobes. Segment 5 with a small medial lobe and two large lateral lobes. Segment 6–9 also with pair of ventral lateral lobes but the size gradually decreased posteriorly. Segment 10 and posterior segments without ventral lateral lobes (Figs 1A, 2A, B). Segment 20 with dorsal posterior lobe.

*Chaetigers* 1–3 (segments 5–7) uniramous with pair of wedge-shaped notopodia only (Fig. 1A–B). Chaetigers 4–16 (segments 8–20) biramous with pair of wedge-shaped notopodia and pair of ear-shaped neuropodia (Fig. 1A–D). Segment 21 with a dorsal posterior lobe and pair of lateral lobes, but without chaetae (Figs 1C–D, 3H–I).

*Notopodia* with two kinds of capillaries forming bundle: both with finely hirsute surface on anteromedian margin and smooth surface on posterior margin; one kind with serrations along anterior portion of tip; the other kind smooth, tapering to very acute tip (Figs 1G–H, 2D–J). Neuropodia with uncini arranged in row along the ridge, each uncinus with major teeth arranged in two rows, 7–8 teeth per row (Figs 1E, 2K).

*Scaphe* distinctly separated from segment 21 (Figs 1A, 3A–B), formed by fusion of five posterior segments. Scaphe longer than broad, arched ventrally and flattened dorsally with crenulated lateral margin (Figs 1B–C, 3D, H–I). Anal flap tongue-shaped with tip extending beyond posterior scaphal edge; anal flap margin crenulated (Figs 1D, 3D–E). Scaphal hooks short, barely visible, with a curved blunt tip; five on right and four on left; present in the dorsolateral region of scaphe, approximately half way between the junction with segment 21 and the posterior edge (Figs 1F, C–D, 3F, I).

#### Variation.

Comparison among the types shows that the body width in the cephalic region varies from 8 to 10 mm. The number of paleae varies from 11 to 12 pairs. The number of cirri on the cephalic veil margin varies from 26 to 32. The number of scaphal hooks varies from 4 to 5 pairs. Since there are only two complete and one incomplete specimens of similar sizes, intraspecific variation amongst these morphological characters may have been underestimated.

#### Type locality and distribution.

Currently only known from Daya Bay (Guangdong Province) and Beibu Gulf (Guangxi Province) in the northern South China Sea.

#### Remarks.

*Pectinaria torquata* sp. n. can be distinguished from the other 25 described species of *Pectinaria* by several features (Table 1). First, it has a dorsal posterior lobe on segment 2, which is unique among the described species of *Pectinaria* species. This structure is prominent in the type specimens, and does not change by preservation. Second, *P. torquata* sp. n. has a dorsal posterior lobe on both segment 20 and 21, a feature that has not been reported from any other recognized species in this genus (Hartman 1941, Long 1973, Hutchings and Peart 2002, Nishi et al. 2014, Wong and Hutchings 2015). Third, *P. torquata* sp. n. has only 4–5 pairs of small blunt scaphal hooks, which is fewer than those in most described species. Fourth, the anal flap lacks a middorsal anal cirrus with a crenulated margin. There are eight species in which the dorsal posterior lobe on segment 2 is not recorded (Table 1). Except for *Pectinaria dimai* Zachs, 1933 and *Pectinaria panava* Willey, 1905 for which the middosal anal cirrus is not described, the other five species have a middorsal anal cirrus; *P. torquata* sp. n. lacks a middorsal anal cirrus (Table 1). Furthermore, it differs from *P. dimai* which has 3–4 rows of major teeth on each uncinus, and 6–7 teeth per row; *P. torquata* sp. n. has 2 rows of major teeth, and 7–8 teeth per row. *Pectinaria panava* has more scaphal hooks than *P. torquata* sp. n.

A key to eleven species of Pectinariidae, including *P. torquata* sp. n., that have been recorded from the South China Sea in the literature is provided below (Wu and Chen 1985, Yang and Sun 1988, Paxton and Chou 2000, Sun and Qiu 2012, Salazar-Vallejo et al. 2014, Zhang et al. 2015, Glasby et al. 2016). The genera *Petta* and *Cistenides* have not been recorded in the South China Sea.

**Table 1.** Major diagnostic characters of *Pectinaria*.

Species	Number of cirri on cephalic veil	Number of pairs of paleae	Posterodorsal lobe in segment 2	Rows of major teeth per uncinus	Middosal anal cirrus of anal flap	Scaphal hooks		Distribution	Literatures cited
						Type	Number of pairs		
*Pectinaria aegyptia* (Savigny, 1818)	60–65	15–17	n.r.	2	present	pointed, strongly curved	4–5,	Red Sea, Japan	Hutchings and Peart 2002, Nishi et al. 2014
*P. antipoda* Schmarda, 1861	17–29	5–13	absent	2–4	present	blunt, curved or spiral	6–8	Australia	Hutchings and Peart 2002, Hutchings 2015
*P. belgica* (Pallas, 1766)	17–28	8–15	absent	2–4	present	pointed	6–12	Sweden, Japan	Hutchings and Peart 2002, Nishi et al. 2014
*P. brevispinis* Grube, 1878	22–30	10–13	absent	2	absent	blunt	8–14	Philippines, Indonesia	Nilsson1928, Hartman 1941
*P. californiensis* Hartman, 1941	18–30	13–14	n.r.	2	present	pointed, slight curved	13	Southern California	Hartman 1941
*P. c. newportensis* Hartman, 1941	19	12–14	n.r.	2	present	pointed, curved	12–13	California	Hartman 1941
*P. carnosus* Wong & Hutchings, 2015	16	9	absent	2	absent	blunt, slight curved	6	Lizard Island	Wong and Hutchings 2015
*P. clava* Grube, 1878	10–12	11	absent	3	n.r.	pointed	6	Lapinig Canal, Philippines	Hutchings and Peart 2002, Nilsson 1928
*P. chilensis* (Nilsson, 1928)	30–60	8–10	n.r.	2	present	slight curved	13–15	Coronel, Chile	Hartman 1941, Moreno et al. 2004
*P. torquata* sp. n.	26–32	11–12	present	2	absent	blunt, curved	4–5	South China Sea	This study
*P. conchilega* Grube, 1878	12	11	absent	3–4	absent	pointed, curved	4	Bohol, Philippines	Nilsson1928
*P. dimai* Zachs, 1933	n.r.	n.r.	n.r.	3–4	n.r.	n.r.	n.r.	North Japan Sea	Zachs1933, Hutchings and Peart 2002
*P. dodeka* Hutchings & Peart, 2002	16–28	11–13	absent	2–4	present	pointed, stongly curved	6–10	Queensland, Australia	Hutchings and Peart 2002
*P. gouldii* (Verrill, 1874)	12–38	9–15	n.r.	2–4	present	Lanciform, pointed, strait or slight curved	8–22	Long Island Sound	Hartman 1941, Long 1973
*P. hartmanae* Reish, 1968	30	8–10	n.r.	2	present	blunt	8–10	California, USA	Reish 1968
*P. hiuchiensis* Kitamori, 1965	32–35	9–10	absent	2	absent	pointed, slight curved	8–9	Kyushu, Japan	Nishi et al. 2014
*P. kanabinos* Hutchings & Peart, 2002	10–16	12–14	absent	2–4	present	pointed, stongly curved	4–6	Queensland, Australia	Hutchings and Peart 2002
*P. longispinis* Grube, 1878	17	13	absent	n.r.	n.r.	n.r.	4	Philippines	Grube 1878, Hutchings and Peart 2002
*P. meredithi* Long, 1973	16–21	8–11	n.r.	2–3	present	pointed, strait or slight curved	7–9	Bahamas and Florida Keys	Long 1973
*P. nana* Wesenberg-Lund, 1949	8	11	absent	n.r.	present	n.r.	3	Gulf of Oman Iran	Wesenberg-Lund 1949
*P. okudai* (Imajima & Hartman, 1964)	10–15	13–16	absent	3–4	present	pointed, slight curved	12–13	Japan	Nishi et al. 2014
*P. panava* Willey, 1905	32	10	n.r.	n.r.	n.r.	n.r.	7	Sri Lanka	Hutchings and Peart 2002
*P. papillosa* Caullery, 1944	24–46	11–13	absent	2	absent	n.r.	3–11	Indonesia	Day 1951
*P. parvibranchis* Grube, 1878	12–13	10–11	absent	3–4	n.r.	pointed	4	Pangloo, Philippines	Nilsson 1928, Hartman 1941, Hutchings and Peart 2002
*P. profunda* Caullery, 1944	20	12	n.r.	2	present	n.r.	n.r.	Indonesia	Hutchings and Peart 2002
*P. regalis* (Verrill, 1901)	21–35	8–14	absent	2–3	present	pointed, strait	0–4	Cony Island, Bermuda	Hartman 1941, Long 1973

n.r. character not recorded.

### Key to eleven species of Pectinariidae from the South China Sea

**Table:** 

1	Opercular rim smooth	**5**
–	Opercular rim cirrate	***Amphictene* (2)**
2	Less than 15 pairs of scaphal hooks	**3**
–	More than 15 pairs of scaphal hooks	**4**
3	5–8 pairs of scaphal hooks; opercular rim with cirri	***Amphictene capensis* (Pallas, 1776)**
–	12 pairs of scaphal hooks; opercular rim with denticles	***Amphictene leioscapha* (Caullery, 1944)**
4	19–24 pairs of scaphal hooks; without pair of dorsolateral lobes on segment 3	***Amphictene japonica* Nilsson, 1928**
–	26–37 pairs of scaphal hooks; with pair of dorsolateral lobes on segment 3	***Amphictene alata* Zhang, Zhang & Qiu, 2015**
5	Cephalic veil laterally attached	***Lagis* (6)**
–	Cephalic veil free	***Pectinaria* (8)**
6	Branchiae absent	***Lagis crenulatus* Sun & Qiu, 2012**
–	Branchiae present	**7**
7	Margins of anal lobe with long fringes	***Lagis bocki* (Hessle, 1917)**
–	Margins of anal lobe without fringes	***Lagis koreni* Malmgren, 1866**
8	Segment 2 with a posterodorsal lobe	***Pectinaria torquata* sp. n.**
–	Segment 2 without a posterodorsal lobe	**9**
9	6–8 pairs of scaphal hooks; anal flap with a small cirrus	***Pectinaria antipoda* Schmarda, 1861**
–	3–4 pairs of scaphal hooks; anal flap without anal cirrus	**10**
10	Cephalic veil with 24–46 cirri; major teeth of uncini in 2 rows	***Pectinaria papillosa* Caullery, 1944**
–	Cephalic veil with 12 cirri; major teeth of uncini in 3–4 rows	***Pectinaria conchilega* Grube, 1867**

## Supplementary Material

XML Treatment for Pectinariidae

XML Treatment for Pectinaria torquata
